# Ultrafast Charge Carrier Dynamics in CuWO_4_ Photoanodes

**DOI:** 10.1021/acs.jpcc.0c11607

**Published:** 2021-03-04

**Authors:** Ivan Grigioni, Annalisa Polo, Maria Vittoria Dozzi, Lucia Ganzer, Benedetto Bozzini, Giulio Cerullo, Elena Selli

**Affiliations:** † Dipartimento di Chimica, Università degli Studi di Milano, Via Golgi 19, 20133 Milano, Italy; ‡ Department of Physics, Politecnico di Milano, IFN-CNR, Piazza Leonardo da Vinci 32, 20133 Milano, Italy; § Department of Energy, Politecnico di Milano, via Lambruschini 4, 20156 Milano, Italy

## Abstract

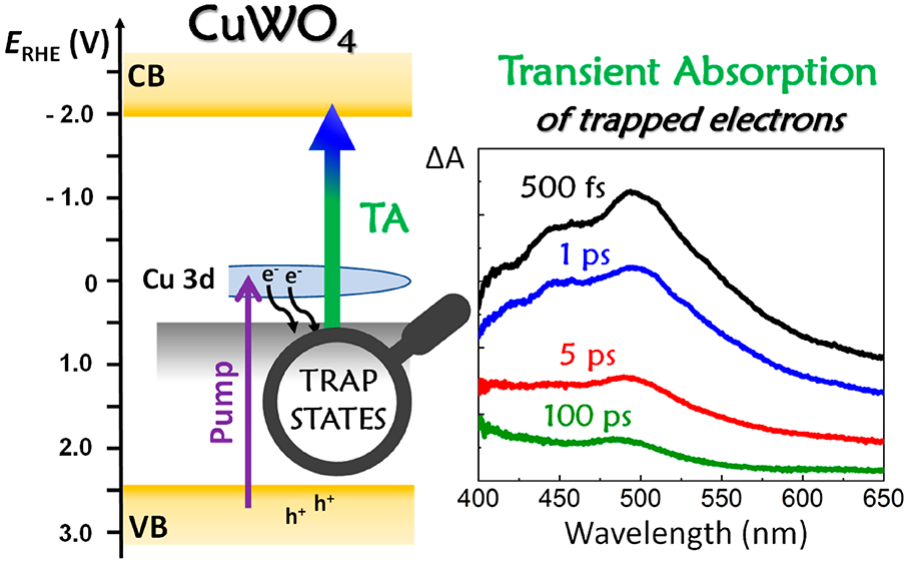

CuWO_4_ is a ternary metal oxide semiconductor with promising properties for photoelectrochemical (PEC) water splitting and solar light conversion, due to its quite low band gap (2.3 eV) and high stability in an alkaline environment. Aiming at understanding the origin of the relatively low PEC efficiency attained with CuWO_4_ photoanodes, we here investigate transparent CuWO_4_ electrodes prepared by a simple solution-based method through the combination of femtosecond transient absorption spectroscopy with electrochemical, PEC, and photochromic characterizations. The very fast recombination dynamics of the charge carriers photogenerated in CuWO_4_, which is the reason for its low efficiency, is discussed in relation with its PEC performance and with the recently calculated band structure of this material, also in comparison with the behavior of other semiconductor oxides employed in PEC applications, in particular Fe_2_O_3_.

## 1 Introduction

Solar energy conversion and storage into fuels such as H_2_ through water splitting form a viable way to achieve a future based on renewable energy sources.^1−3^ The photocatalytic approach is most promising among those allowing one to transform intermittent and abundant solar light into valuable energy vectors. In this regard, the photocatalytic water splitting technology and, in particular, photoelectrochemical (PEC) tandem cells in which two light harvesting materials with band gaps matching two different portions of the visible spectrum are coupled would in principle allow a solar light energy conversion efficiency up to 31%.^4−6^ For PEC water oxidation purposes, visible light active, stable, and earth abundant materials are highly desirable.^7^ Metal oxide semiconductors are very attractive for this application, being intrinsically more stable toward oxidation than other visible light harvesting materials.^8^

TiO_2_, WO_3_, Fe_2_O_3_, and BiVO_4_ are the most studied photoactive materials. All of them present advantages in many respects but also drawbacks.^6,8^ In particular, Fe_2_O_3_ has a band gap of 2.1 eV, which is low enough to allow a solar light conversion efficiency higher than 12%,^9^ but it is characterized by a very fast charge carrier recombination, which seriously limits its performance. BiVO_4_, on the other hand, has longer-lived charge carriers, compatible with an internal quantum efficiency (IQE) close to unity up to 2.75 eV (450 nm), but with its band gap of 2.4 eV, it would theoretically allow a sunlight to hydrogen conversion efficiency of ca. 8% only.^10−12^

Copper tungstate (CuWO_4_), with a band gap of ca. 2.3 eV corresponding to an absorption edge of 550 nm, represents a good candidate material for PEC devices.^13^ It shows good stability in prolonged PEC water oxidation experiments under simulated solar irradiation in neutral and slightly basic solutions.^13^ However, although the exploitation of CuWO_4_ as photoanode material dates back to pioneering works of the 1980s,^14,15^ and despite the fact that it features quantitative selectivity to water oxidation and hole collection at the electrode/electrolyte interface,^13,16,17^ the highest photocurrent obtained with CuWO_4_-based electrodes is only 0.2–0.3 mA cm^–2^ at 1.23 V_RHE_,^18,19^ as compared to, e.g., 6.7 and 6 mA cm^–2^ obtained with state of the art BiVO_4_ and Fe_2_O_3_ photoanodes.^20,21^ The reasons for such a poor PEC performance are unclear, also because the dynamics of the charge carriers photogenerated in CuWO_4_ is still unexplored.

Here we investigate the ultrafast dynamics of the charge carriers photogenerated in transparent CuWO_4_ electrodes through femtosecond transient absorption (TA) spectroscopy and discuss it in relation to the recently proposed electronic structure of CuWO_4_, which receives full support from the here performed PEC characterization of this ternary oxide.

## 2 Methods

### 2.1 Materials

The following chemicals, all purchased from Sigma-Aldrich, were employed in the present work: copper(II) nitrate trihydrate 99%, citric acid 99%, tetrabutylammonium hexafluorophosphate 99%, nitric acid 65%, anhydrous sodium sulfite, and boric acid ACS reagent, together with ammonium tungsten oxide hydrate 99% (Fluka). Deionized Millipore water was employed to prepare the solutions.

### 2.2 Photoelectrode Preparation

A 0.5 M solution of CuWO_4_ was prepared as follows. First 14 mmol of citric acid were dissolved in 5.3 mL of ethanol and 2.4 mL of deionized H_2_O. Then 5 mmol of Cu(NO_3_)_2_·3H_2_O and 0.4167 mmol of (NH_4_)_6_H_2_W_12_O_40_·*x*H_2_O were sequentially added, under stirring. A film was grown from this solution onto fluorine-doped tin oxide (FTO) glass (Pilkington Glass, TEC-7, thickness 2 mm) by spin coating at 2000 rpm for 20 s. Prior to deposition, the FTO glass was cleaned by 30 min long sonication in a soap solution, then thoroughly rinsed with water, sonicated for 30 min in ethanol, and finally dried in air. Before moving the FTO slice to the spin coater, it was soaked in 2-propanol for a few seconds. This procedure was found to increase the optical transparency of the final film. After spin coating, the CuWO_4_ film was preannealed at 250 °C for 10 min and then annealed at 550 °C for 1 h.

### 2.3 Optical, Morphological, and Structural Characterization

**Figure 1:**
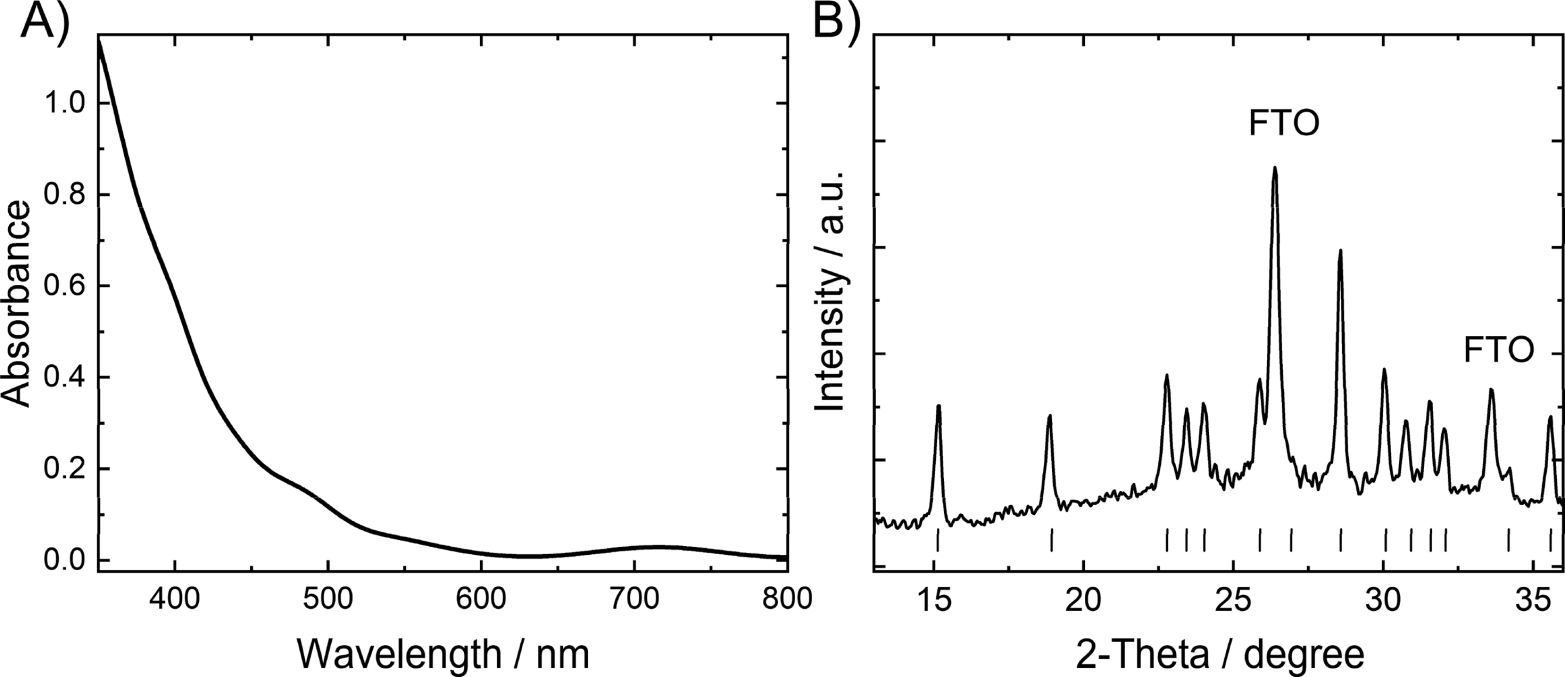
(A) Absorption spectrum of a CuWO_4_ photoelectrode. (B) X-ray diffraction pattern of the CuWO_4_ thin film coated on FTO glass. The gray bars are the reference Bragg reflections of CuWO_4_ from JCPDF 72-0616. The FTO reflections are marked separately.

The absorption spectra were recorded in the transmission mode with a Jasco V-650 spectrophotometer. The spectrum shown in Figure 1A refers to a double layered CuWO_4_ film electrode, to minimize the presence of interference fringes. Top view and cross-section scanning electron microscopy (SEM) images were acquired by employing a LEO 1430 scanning electron microscope operating at a 10 kV accelerating voltage and 8 mm working distance. The crystalline phase of the material was determined through X-ray diffraction (XRD) analysis using a Philips PW 1830/40 instrument, equipped with a Cu sealed tube at 40 kV and 40 mA.

### 2.4 Photoelectrochemical Measurements

PEC measurements were performed in a homemade three electrode cell equipped with two quartz windows. The FTO/CuWO_4_ electrode was used as working electrode, a Pt wire as counter electrode, and a Ag/AgCl electrode as reference electrode. The photoanodes were tested under back (from the FTO side) irradiation. The light source was an Oriel, Model 81172 solar simulator equipped with a AM 1.5 G filter. The light intensity, measured by means of a Thorlabs PM200 power meter equipped with a S130VC power head with Si detector, was 100 mW cm^–2^. PEC measurements were carried out with the electrode in contact with a 0.1 M buffer borate (KBi) solution at pH 9 or with 0.1 M pH 9 KBi containing 0.5 M Na_2_SO_3_.

The KBi solution was prepared by dropwise adding a 1.0 M KOH solution to aqueous boric acid up to pH 9, followed by dilution to 0.1 M. Prior to performing the linear scans, the open circuit potential (OCP) of the electrode was measured for 10 min under irradiation. Then the scan started from the so determined OCP with a 10 mV s^–1^ sweep rate. The potential vs Ag/AgCl was converted to the RHE scale at pH 0 (corresponding to NHE) using the following equation: *E*_RHE_ = *E*_Ag/AgCl_ + 0.059 pH + *E*°_Ag/AgCl_, with *E*°_Ag/AgCl(3.0 M NaCl)_ = 0.210 V at 25 °C.

Incident photon to current efficiency (IPCE) scans were recorded under a 1.23 V vs RHE bias, and the current was measured with a 10 nm step, within the 300–550 nm wavelength range. A 300 W Lot-Oriel Xe lamp was employed as an irradiation source, equipped with a Lot-Oriel Omni-λ 150 monochromator and a Thorlabs SC10 automatic shutter. The incident light power was measured at each irradiation wavelength, and the IPCE was calculated as
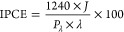
1where *J* (A cm^–2^) is the photocurrent density and *P*_*λ*_ (W cm^–2^) is the power of the monochromatic light at the wavelength λ (nm). The internal quantum efficiency (IQE) was calculated by combining the IPCE curve with the absorption (*A*) spectrum of the photoanode, as follows:
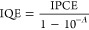
2

### 2.5 Photochromic Measurements

The photochromic experiments were performed by irradiating a 25 × 9 mm^2^ CuWO_4_ electrode, sealed inside a N_2_ filled glovebox in a 10 mm-thick cuvette modified to allow the insertion of the 9 mm wide electrode and containing 3 mL of ethanol previously stored overnight in the glovebox, in order to attain a completely oxygen free environment. The film was irradiated with a 300 W Xe arc lamp (Lot Oriel, LSH302), with a light intensity of ca. 150 mW cm^–2^, measured with a PM100A Thorlabs power meter equipped with a S302C thermal power sensor. The photochromic effect was monitored by recording the absorption spectra at fixed irradiation times with a Shimadzu model 3600 UV–vis–IR spectrophotometer. The irradiated cuvette was cooled with a flux of compressed air.

At the end of the experiment, the CuWO_4_ electrode in the airtight cuvette was moved to the spectrophotometer, the cuvette was opened to air, and the subsequent absorption spectrum changes were monitored. Almost complete CuWO_4_ back oxidation was attained after 11 h long contact with air.

### 2.6 Electrochemical Measurements

The Mott–Schottky plot was obtained by acquiring a series of potentiostatic electrochemical impedance spectroscopy (EIS) spectra employing a Parstat potentiostat, with a sinusoid of peak-to-peak amplitude of 10 mV in the 10^5^–0.1 Hz frequency range. The electrolyte employed was a 0.3 M Na_2_SO_4_ solution at pH 7. The bias ranged between −0.2 and +0.6 V vs Ag/AgCl (V_Ag/AgCl_), and the potentials were scanned in the cathodic direction, to avoid possible irreversibility due to reduction of the metallic components. Capacitance values were extracted by a nonlinear least-squares fitting with an equivalent-circuit model featuring the parallel of a faradaic resistance and a double-layer capacitance, in series with an ohmic resistance (Randle’s model).^16^ As is customary in EIS modeling, morphological heterogeneities of the film were accounted for by replacing the double-layer capacitance with a Constant Phase Element (CPE). Fitting was carried out with a Matlab-based program written by the authors, allowing better control over the regression statistics than the commonly used commercial packages. The CPE exponent estimates ranged in the 0.86–0.98 interval and did not exhibit any measurable potential dependence. The extrapolation to zero of the linear part of the Mott–Schottky plot^22^ and the subsequent correction of the intercept for *kT*/q ≈ 25 mV at room temperature, provided a value of 0.51 V_RHE_.

Cyclovoltammetry experiments were performed by polarizing a 25 × 9 mm^2^ electrode within the same cuvette employed in the photochromic experiments. The ohmic contact was obtained by soldering with indium a copper wire to the uncovered FTO edge; the contact was then glued with an epoxy resin (Araldite) for insulation. Two platinum wires were used as counter and quasi-reference electrodes, respectively, and the cuvette was closed with a rubber septum. The potential was modulated with an Autolab PGSTAT 12 instrument (EcoChemie, Utrecht, The Netherlands) controlled by the NOVA software. The potential of the Pt pseudoreference electrode was calibrated with respect to RHE using the ferrocene/ferrocenium redox couple (+400 mV_RHE_) as standard.^23^ The electrolyte was a 0.1 M tetrabutylammonium hexafluorophosphate solution in acetonitrile, which was deaerated through N_2_ bubbling for 5 min prior to the electrochemical experiments.

### 2.7 Transient Absorption Characterization

Femtosecond transient absorption (TA) experiments were performed using an amplified Ti:sapphire laser system (Libra, Coherent) delivering 4 mJ, 100 fs pulses at wavelength λ = 800 nm with 1 kHz repetition rate. A 400-μJ fraction of the laser energy was used for the experiment, 95% of which was frequency doubled to λ = 400 nm and used as a pump, while the remaining 5% was focused on a CaF_2_ crystal to generate the white light continuum probe, covering the 380–680 nm wavelength range. Alternatively, the output of an optical parametric amplifier was used to generate the pump pulses at λ = 500 nm. TA spectra were measured with an Acton Sp2150 spectrometer (Princeton Instruments) equipped with a CCD camera (Entwicklungsbro Stresing) detecting data at the full 1 kHz repetition rate of the laser. The photoexcited spot area was 6 × 10^–4^ cm^2^ with a pump energy ranging from 16 to 500 nJ, corresponding to a fluence from ca. 27 to 830 μJ cm^–2^.

## 3 Results and Discussion

### 3.1 Characterization of the CuWO_4_ Photoanodes

As shown in Figure 1A, the CuWO_4_ photoanodes prepared by spin coating a precursor solution on FTO, followed by annealing, are optically transparent at wavelengths longer than the absorption edge of the material. The absorbance drops above 350 nm, due to the indirect band gap and the low absorption coefficient of CuWO_4_;^22^ the broad and weak feature above 700 nm is the tail of the Cu d–d transition.^24^

The X-ray diffraction pattern of a polycrystalline CuWO_4_ film, shown in Figure 1B, coincides with the set of Bragg reflections reported in reference JCPDF 72-0616, without reflections typical of WO_3_, CuO, and Cu_2_O, indicating that CuWO_4_ is in pure triclinic form.^15,16,22^

**Figure 2:**
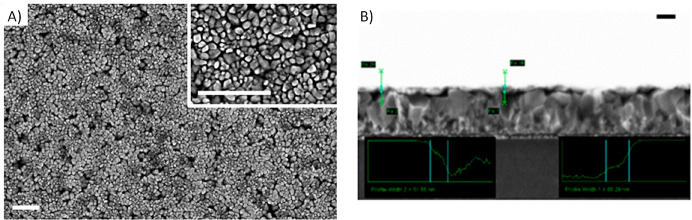
(A) SEM top view and (B) cross section image of the CuWO_4_/FTO electrode employed in the PEC measurements. The scale bars are 500 (A) and 200 nm (B).

The top view SEM image presented in Figure 2A confirms that the CuWO_4_ layer completely covers the conductive FTO substrate and is composed of small grains with ca. 50 nm average diameter. The CuWO_4_ film in the here investigated electrodes is ca. 80 nm thick, as estimated from the cross section SEM image shown in Figure 2B.

**Figure 3:**
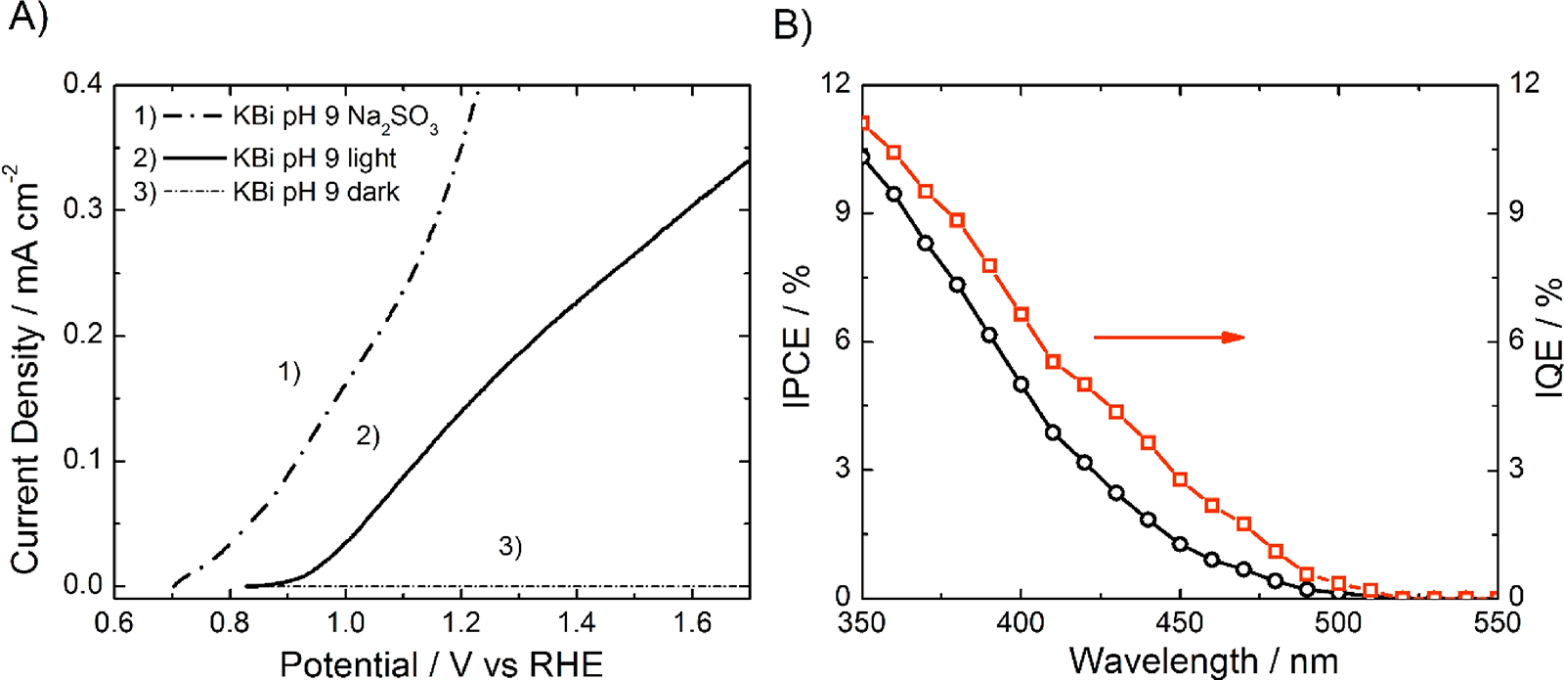
(A) Linear sweep voltammetry under AM 1.5 G simulated solar light for the CuWO_4_ film in contact with 0.1 M potassium borate (KBi) aqueous solutions at pH 9, (1) in the presence of 0.5 M Na_2_SO_3_ (dash dot line) and (2) in the absence of Na_2_SO_3_ (continuous line) and (3) in the absence of irradiation and of Na_2_SO_3_. (B) Incident photon to current efficiency (IPCE, black circles) and internal quantum efficiency (IQE, red squares) of the CuWO_4_ photoanode at 1.23 V_RHE_ in KBi at pH 9.

Figure 3A reports the linear sweep voltammetry recorded with the CuWO_4_ electrode in contact with a 0.1 M potassium borate aqueous solution buffered at pH 9, both in the presence and in the absence of 0.5 M Na_2_SO_3_ as hole scavenger. The photocurrent values obtained at 1.23 V_RHE_ in the presence or absence of sulfite, i.e., 0.40 and 0.15 mA cm^–2^, respectively, are compatible with those recorded with electrodes prepared through other routes.^16,19,22^

As expected, in the presence of an electron donor the photocurrent onset potential shifts to more negative values.^25^ However, only a ca. 3-fold photocurrent density increase was observed at 1.23 V_RHE_ upon addition of Na_2_SO_3_ as a hole scavenger, which is 2 orders of magnitude smaller than that measured, for instance, with BiVO_4_ at 1.23 V_RHE_ in the presence of the same hole scavenger.^26^ This supports the view that surface water oxidation is almost quantitative on CuWO_4_, as recently outlined by Gao et al.,^27^ and it implies that the overall efficiency of this material is limited by poor internal charge transport or fast bulk charge carrier recombination, hampering efficient hole diffusion from the excitation site to the oxide surface.

The incident photon to current efficiency (IPCE) curve shown in Figure 3B reflects the absorption spectrum of CuWO_4_ (see Figure 1A), decreasing with increasing wavelength. The IQE curve, obtained by normalizing the photocurrent for the intensity of absorbed light, has a similar wavelength dependence. The photocurrent onset is at ca. 510 nm, in line with that reported for bulk CuWO_4_^16,28^ and slightly red-shifted with respect to that of WO_3_ (absorption onset at ca. 480 nm).^29^

**Figure 4:**
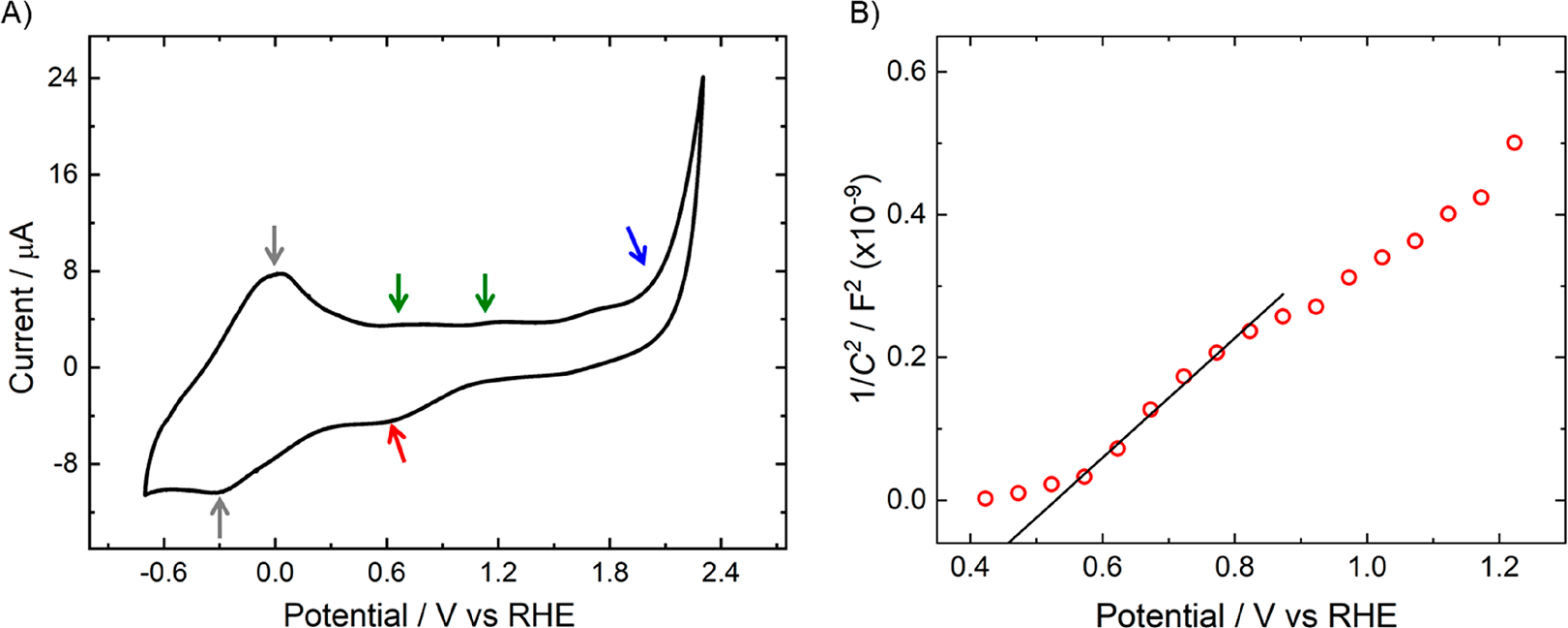
(A) Cyclovoltammetry recorded in 0.1 M tetrabutylammonium hexafluorophosphate, with a scan rate of 100 mV s^–1^. (B) Mott–Schottky plot of the CuWO_4_ photoanode in 0.3 M Na_2_SO_4_ solution at pH 7.

A cyclovoltammetry (CV) test was employed to investigate and localize redox states within the band gap of CuWO_4_. As shown in Figure 4A, a Butler–Volmer type current rise (marked with a blue arrow), starting at ca. 2.0 V_RHE_, indicates the oxidation of the highest occupied states of the valence band (VB) of CuWO_4_. At the lowest investigated potential range a quasi-reversible redox process centered at ca. 0 V_RHE_ (cathodic and anodic peaks marked with gray arrows) is found, which might be related to the reduction and oxidation of the narrow band composed of localized Cu 3d states, as first proposed by Pyper et al.^30^ and confirmed by recent calculations.^31^

The voltammetric features found in the potential range between the VB oxidation threshold (ca. 2.0–2.4 V_RHE_) and the Cu 3d states (ca. 0 V_RHE_), consisting of two relatively weak oxidation peaks (marked with green arrows in Figure 4A) and a broad cathodic feature (marked with a red arrow), can thus be interpreted as the redox response of intragap states, possibly located in the 0.5–1 V_RHE_ range.

The Mott–Schottky plot recorded with the CuWO_4_ photoanode in 0.3 M Na_2_SO_4_ solution at pH 7 (Figure 4B) presents a change in slope that suggests the occurrence of Fermi level pinning.^30^ This can be related to the presence of midgap states, energetically located at 0.51 V_RHE_ minimum potential, as calculated from the intercept of the Mott–Schottky plot on the abscissa axis (see Section 2.3), and perfectly in line with CV results.

**Figure 5:**
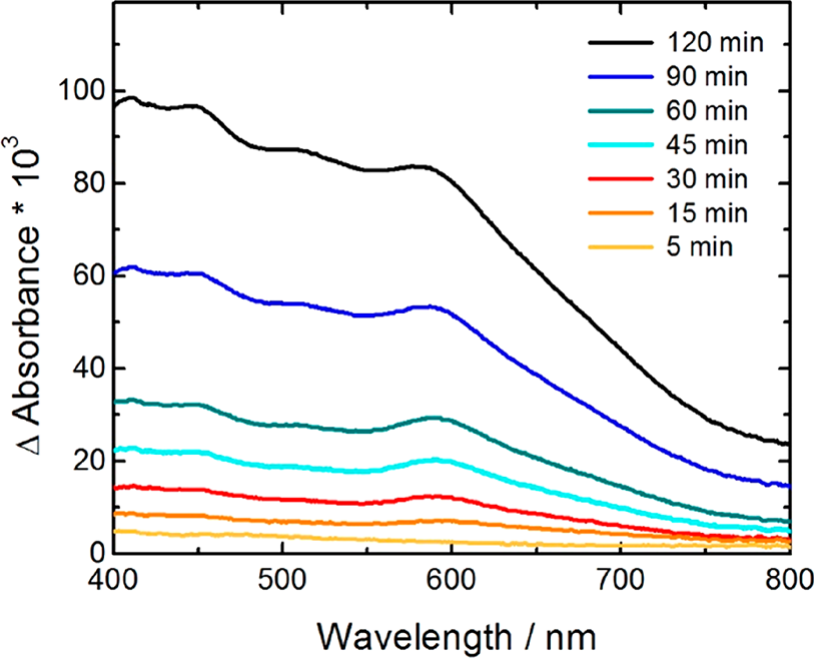
Photochromic measurements of a CuWO_4_ electrode recorded in deaerated ethanol at increasing time under irradiation with a 300 W Xe arc lamp.

The photochromic behavior of CuWO_4_ was investigated by irradiating a film in deaerated ethanol, followed by monitoring the absorption spectrum changes by spectrophotometric analysis. Figure 5 shows that a positive absorption variation Δ*A* was observed, extending over the visible spectrum with a shoulder at ca. 600 nm, which linearly increased under irradiation.

This signal can be assigned to electrons trapped in CuWO_4_, in line with the Δ*A* signal decrease with time observed when, after 2-h-long irradiation, the film was exposed to air (see Figure S1 in the Supporting Information). Thus, evidence is obtained that electron trapping in CuWO_4_ is reversible and that metastable trapped states can be back-oxidized by atmospheric oxygen.

### 3.2 Femtosecond Transient Absorption Spectroscopy

**Figure 6:**
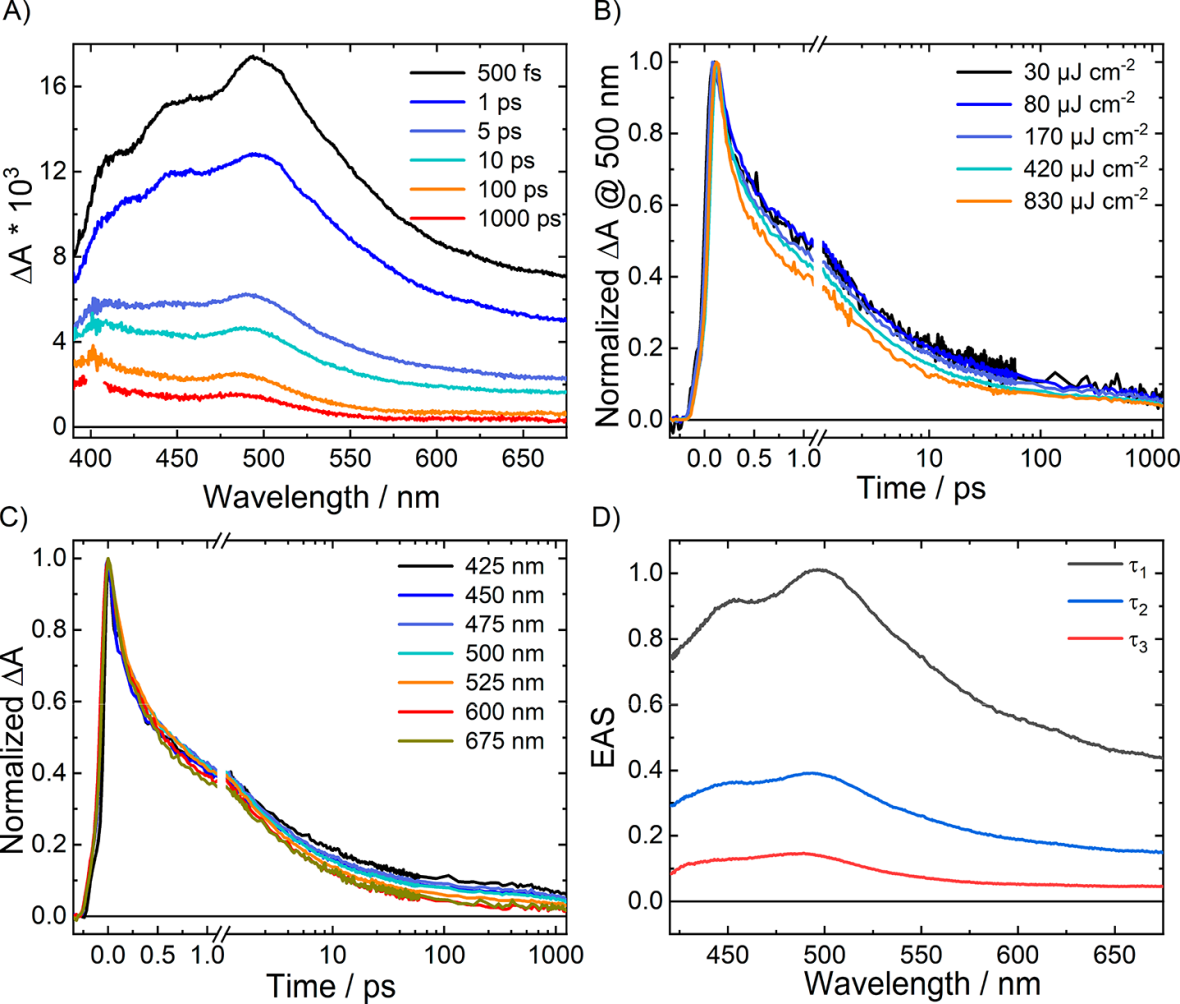
(A) Femtosecond TA spectra recorded at different times after photoexcitation of CuWO_4_ at 400 nm under vacuum and Δ*A* decay traces (B) at 500 nm upon excitation with different pump fluences and (C) at different wavelengths upon excitation with a 420 μJ cm^–2^ fluence. (D) Evolution associated spectra (EAS) obtained from Global Analysis of the Δ*A*(λ,Δ*t*) data recorded upon photoexcitation at 400 nm with a 170 μJ cm^–2^ fluence.

The ultrafast charge carrier dynamics in CuWO_4_ was here investigated through femtosecond TA spectroscopy. Previously, only a time-resolved microwave conductivity investigation was reported on the long-lived charge carriers in CuWO_4_.^32^ Upon excitation at 400 nm, promoting electrons from the VB of CuWO_4_ into the localized Cu 3d-based band, the film exhibits a broad transient absorption increase (Δ*A* > 0) extending over the entire visible spectral range (Figure 6A), similar to the Δ*A* feature recorded in photochromic measurements (Figure 5), which is thus ascribed to trapped electrons. The results are similar to those obtained with Fe_2_O_3_ oxide in the same time scale.^33,34^ In the case of CuWO_4_, the transient spectrum does not present any photobleaching (Δ*A* < 0).

In order to investigate how transient decay processes depend on the density of photogenerated charges, TA experiments were performed at different excitation fluences in the 27 to 830 μJ cm^–2^ range. The Δ*A* values monitored at different wavelengths increase linearly with the pump fluence (Figure S2), indicating that multiphoton absorption of the pump and nonlinear carrier interactions are not significantly contributing to the observed signal.

The decays of Δ*A* signal obtained with different pump fluences and monitored at 500 nm are reported in Figure 6B. No significant change in the dynamics can be appreciated upon varying the pump fluence, within the investigated fluence range. This indicates that higher order kinetics, like a carrier–carrier annihilation (Auger recombination) decay mechanism, does not play any relevant role.

The TA signal in CuWO_4_ decays much faster than in other semiconductor oxides, with a behavior similar to that observed in Fe_2_O_3_.^33^ The normalized Δ*A* time traces at different probe wavelengths, shown in Figure 6C, indicate that the signal halves in ca. 1 ps, with only a slight dependence on the detection wavelength. The Δ*A* decay becomes slightly faster by moving the monitoring wavelength from 425 to 600 nm, without any appreciable difference at wavelengths above 600 nm. The lack of a strong dependence of the dynamics on the probe wavelength suggests that the carriers are trapped in the Cu 3d band or in lower energy midgap states within the pump laser pulse, in a way similar to that reported in pioneering ultrafast TA studies on hematite.^33^

In order to shed light on the overall evolution of the Δ*A* signal as a function of wavelength and delay, and to better visualize individual spectral contributions over the entire probe wavelength range, Global Analysis^35,36^ was carried out on the data set acquired with a representative pump fluence of 170 μJ cm^–2^. The time-dependent TA spectra reported in form of evolution-associated spectra (EAS) in Figure 6D consist of three kinetic components with different time constants, which span over the entire investigated 400–700 nm wavelength region. The analysis of their relative amplitudes indicates that the fastest component, with a time constant τ_1_ = 780 ± 10 fs, dominates over the entire spectral range. The rest of the Δ*A* signal decays with a time constant τ_2_ = 9.4 ± 0.1 ps, with only a small contribution from the longest time constant τ_3_ = 1.18 ± 0.02 ns.

In agreement with the single decay traces reported in Figure 6C, the results of Global Analysis are in line with a slight dependence of the signal decay on the monitoring wavelength, with both EAS relative to the first two time constants τ_1_ and τ_2_ exhibiting a maximum around 500 nm and that associated with the slowest component (τ_3_) being slightly blue-shifted and peaking at 480 nm.

A substantially unchanged photoexcitation scenario is observed when pumping closer to the bandgap energy of the material, at 500 nm (2.48 eV). Despite the lower TA signal due to the reduced absorption cross section, the obtained dynamics, reported in Figure S3, is very similar to the one measured upon excitation at 400 nm. Global analysis of the TA data retrieves three components with time constants τ_1_ = 785 ± 10 fs, τ_2_ = 9.3 ± 0.1 ps, and τ_3_ = 1.97 ± 0.02 ns, very close to those obtained upon pumping at 400 nm.

**Scheme 1:**
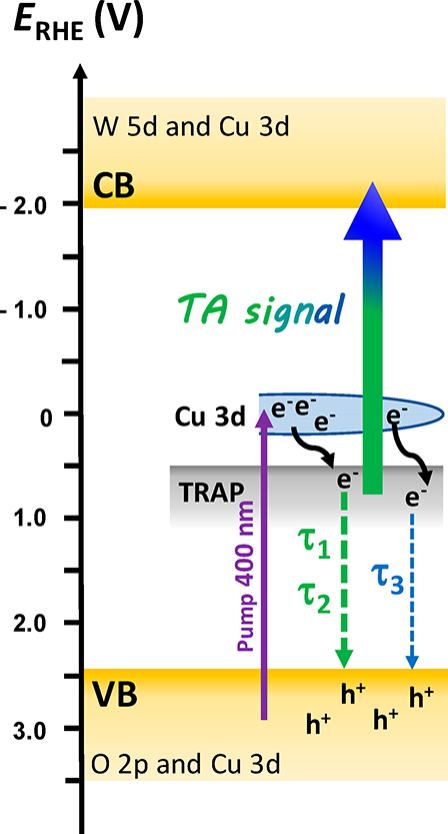
Ultrafast Relaxation and Recombination Pathways of Electrons Photopromoted in CuWO_4_ upon 400 nm Excitation

Our interpretation of TA data well reflects the CuWO_4_ band structure recently calculated by Thang et al.,^31^ by treating the material as a Mott–Hubbard magnetic insulator, a feature common to both CuWO_4_ and Fe_2_O_3_ oxides. According to this model, shown in Scheme 1, the VB of CuWO_4_, consisting of O 2p states partly mixed with Cu 3d states, is separated by ca. 2.1 eV from a narrow band consisting of very localized, Cu empty 3d levels, while a wider band dominated by W 5d and Cu 3d states is located at higher energy, corresponding to a gap of about 5 eV from the top of the VB. Similarly, the recently calculated electronic structure of hematite consists of two energetically well separated conduction bands, both primarily of Fe 3d character, accounting for the detected positive TA signal in the ultrafast regime.^34^

In this picture, the optical absorption in CuWO_4_ associated with the 2.3 eV experimental band gap implies the transition from a not fully localized mixed O 2p–Cu 3d state within the VB to localized Cu 3d states. The localized nature of these Cu 3d states is responsible for the low charge carrier mobility in excited CuWO_4_, while the forbidden character of this d–d transition results in a low absorption coefficient in the visible region.^37^ Furthermore, on the basis of the here collected results by means of different techniques, trap states experimentally detected in the 0.5–1.0 V_RHE_ range are located just below the Cu 3d band

According to Scheme 1, upon ultrafast photoexcitation at 400 nm (3.1 eV), corresponding to an energy just higher than the first localized (Cu 3d in character) band, electrons are promoted from the VB to high energy states of the Cu 3d band, from which they quickly relax within the laser pulse to its bottom or toward midgap states (located just below the Cu 3d band edge).^33,38^ The observed TA signal is thus ascribed to the photoinduced absorption (Δ*A* > 0) of electrons trapped in such states, which are promoted to the W 5d-based high energy conduction band (CB) (Scheme 1).

The above attribution is in agreement with that recently reported for Fe_2_O_3_ investigated in the same ultrafast time scale^34^ and matches well with the maximum absorption energy of the involved transition, located at around 500 nm (≈2.5 eV, see TA spectra in Figure 6A), corresponding to the energy difference between the midgap trap states at around 0.5–1.0 V_RHE_ and the highest empty CB located at ca. −2 V_RHE_ (i.e., 4–5 eV higher than the top of the VB).^31^ Moreover, the ultrafast TA spectra are similar to those recorded in photochromic tests in the presence of a hole scavenger, which is able to fill the holes photogenerated in the VB, thus stabilizing the electrons trapped in midgap states. This rules out any contribution to the TA signal originated by the holes photogenerated in the VB.^34^

The fastest time constants τ_1_ and τ_2_ are associated with the recombination with VB holes of electrons located at a distribution of levels in the high-energy range of the midgap states, while the longest time constant τ_3_ is associated with the recombination of electrons trapped more in depth in these midgap states, from which the promotion to the highest W 5d CB requires higher photon energy. This is reflected by the slight blue-shift of the EAS spectrum with a τ_3_ time constant compared to that of the other two components (Figure 6D). This relatively longer-lived, though minority, decay component, associated with deeply trapped electrons, may significantly reduce electron/hole recombination losses favoring hole transfer in water oxidation at the electrode surface.

Massive internal recombination thus appears to be common to both CuWO_4_ and Fe_2_O_3_.^31,39^ Indeed, for both CuWO_4_ and Fe_2_O_3_ the TA spectrum initially decays very fast, compared to other oxides such as TiO_2_ or BiVO_4_.^40−42^ In early femtosecond-TA studies on Fe_2_O_3_, the photoinduced TA signal was found to disappear within 300 ps,^33^ and the lack of long-lived components in Fe_2_O_3_ was reflected by the extremely low photocurrent values (10 μA cm^–2^) reported in pioneering studies on this material.^43^ However, recently investigated nanostructured and doped Fe_2_O_3_-based electrodes exhibit longer-living components and higher efficiencies,^41,44−46^ and a similar improvement in performance might be envisaged also in the case of CuWO_4_.

## 4 Conclusions

In consideration of the higher photocurrent values measured with CuWO_4_ in recent studies^22,27^ and of the encouraging effects recently obtained, for instance, by CuWO_4_ nanostructuring^47^ and doping^48^ or substituting Mo^6+^ for W^6+^ ions,^49^ CuWO_4_ and other cuprates merit further attention as materials for efficient PEC water splitting and other photocatalytic applications, although several limitations need to be carefully addressed in order to enhance their efficiency. On the basis of the large similarity between CuWO_4_ and Fe_2_O_3_, the use of strategies such as doping, nanostructuring, and the combination with proper oxygen evolution catalysts, which largely contributed to increasing the performance of hematite photoanodes,^50^ might lead to enhanced efficiency also in the case of CuWO_4_ photoanodes.

## Supporting Information Available

Reversible photochromism of photoreduced CuWO_4_, fluence dependence of fs-TA, and TA experiments upon pumping at 500 nm (PDF)
