# Supplementary material for: Ultrafast Charge Carrier Dynamics in CuWO4 Photoanodes
Source: J Phys Chem C Nanomater Interfaces. 2021 Mar 4;125(10):5692–9. doi: 10.1021/acs.jpcc.0c11607 (PMC8765008; doi:10.1021/acs.jpcc.0c11607)
Supplement: Supplementary file 1 — jp0c11607_si_001.pdf [file jp0c11607_si_001.pdf]

SUPPORTING INFORMATION

Ultrafast Charge Carrier Dynamics in $CuWO_4$ Photoanodes

Ivan Grigioni,[a,⊥] Annalisa Polo,[a,⊥] Maria Vittoria Dozzi,[a] Lucia Ganzer,[b] Benedetto Bozzini,[c] Giulio Cerullo,[b] and Elena Selli[a,*]

[a] Dipartimento di Chimica, Università degli Studi di Milano, Via Golgi 19, 20133 Milano, Italy

[b] IFN-CNR, Department of Physics, Politecnico di Milano, Piazza Leonardo da Vinci 32, 20133 Milano, Italy

[c] Department of Energy, Politecnico di Milano, via Lambruschini 4, 20156 Milano, Italy

⊥ I.G. and A.P. contributed equally.

* Corresponding Author, elena.selli@unimi.it

1. Reversible photochromism of photoreduced $CuWO_4$

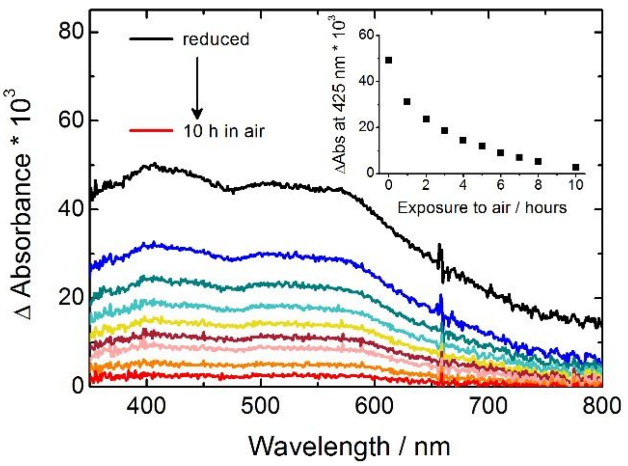

Figure S1. Recovery, upon exposure to air, of the spectral changes induced by $CuWO_4$ irradiation in ethanol under anaerobic conditions; the inset shows the decay of the signal at 425 nm. The here reported difference absorption spectra were obtained by subtracting the spectrum recorded after 11 h exposure to air from the spectra recorded at shorter times.

2. Fluence dependence of fs-TA in $CuWO_4$

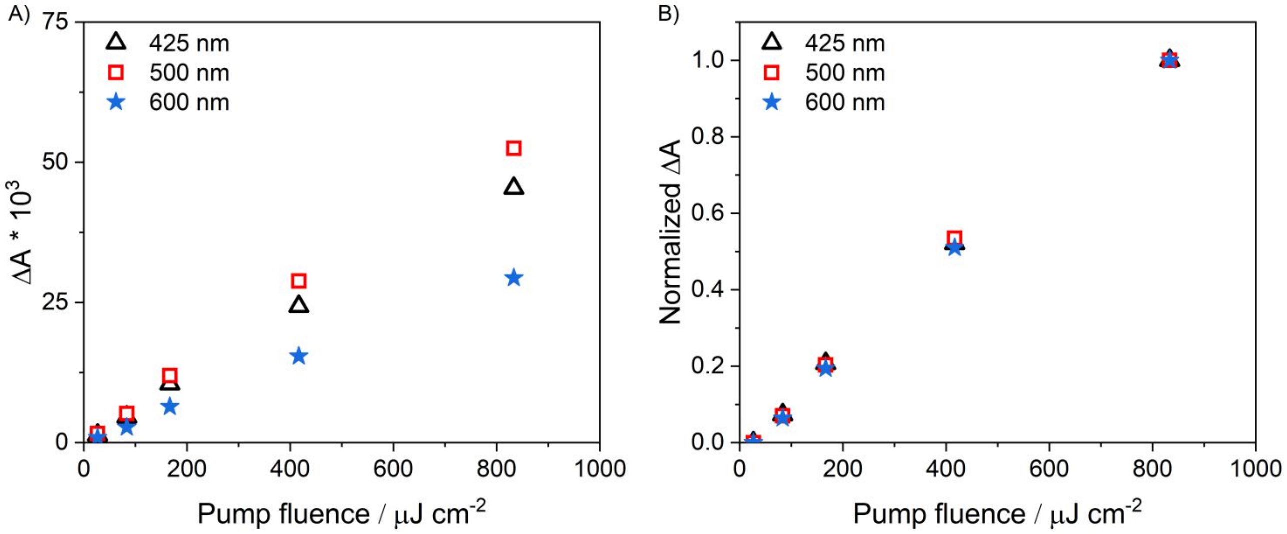

Figure S2. (A) Dependence of the Δ*A* values at 425, 500 and 600 nm on the intensity of the pump fluence; (B) normalized Δ*A* values reported in panel A.

3. TA experiments with pump at 500 nm

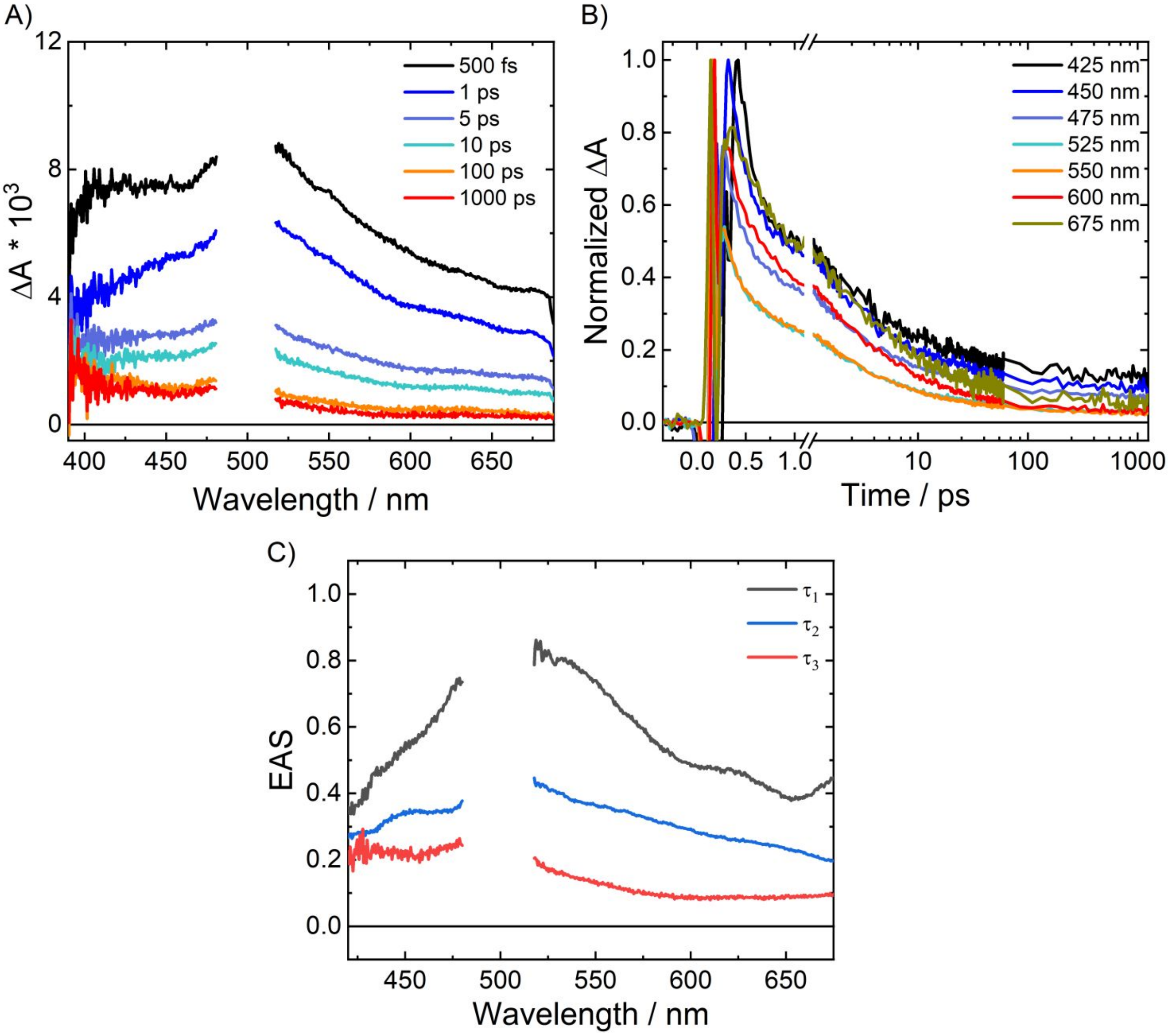

Figure S3. (A) Femtosecond TA spectra recorded at different times after photoexcitation of $CuWO_4$ at 500 nm under vacuum and (B) Δ*A* decay time traces at different wavelengths upon excitation with a 1100 μJ cm^{-2} fluence. (C) Evolution Associated Spectra (EAS) obtained from global analysis of the $\Delta A(\lambda,\Delta t)$ data recorded upon photoexcitation at 500 nm with a 1100 μJ cm^{-2} fluence. The obtained time constants are: τ_1 = 785 ± 10 fs, τ_2 = 9.3 ± 0.1 ps, τ_3 = 1.97 ± 0.02 ns. Due to scattering, the data around the 500 nm pump wavelength are omitted.
